# Activity-dependent formation of a vesicular inhibitory amino acid transporter gradient in the superior olivary complex of NMRI mice

**DOI:** 10.1186/s12868-017-0393-9

**Published:** 2017-10-26

**Authors:** Lena Ebbers, Maren Weber, Hans Gerd Nothwang

**Affiliations:** 1 Neurogenetics Group, Center of Excellence Hearing4All, School of Medicine and Health Sciences, Carl von Ossietzky University Oldenburg, 26111 Oldenburg, Germany; 2 Research Center for Neurosensory Science, Carl von Ossietzky University Oldenburg, 26111 Oldenburg, Germany

**Keywords:** Activity-dependent, Auditory brainstem, Claudin 14, LSO, NMRI, SPN, Tonotopy, Deafness, VGAT, VIAAT

## Abstract

**Background:**

In the mammalian superior olivary complex (SOC), synaptic inhibition contributes to the processing of binaural sound cues important for sound localization. Previous analyses demonstrated a tonotopic gradient for postsynaptic proteins mediating inhibitory neurotransmission in the lateral superior olive (LSO), a major nucleus of the SOC. To probe, whether a presynaptic molecular gradient exists as well, we investigated immunoreactivity against the vesicular inhibitory amino acid transporter (VIAAT) in the mouse auditory brainstem.

**Results:**

Immunoreactivity against VIAAT revealed a gradient in the LSO and the superior paraolivary nucleus (SPN) of NMRI mice, with high expression in the lateral, low frequency processing limb and low expression in the medial, high frequency processing limb of both nuclei. This orientation is opposite to the previously reported gradient of glycine receptors in the LSO. Other nuclei of the SOC showed a uniform distribution of VIAAT-immunoreactivity. No gradient was observed for the glycine transporter GlyT2 and the neuronal protein NeuN. Formation of the VIAAT gradient was developmentally regulated and occurred around hearing-onset between postnatal days 8 and 16. Congenital deaf *Claudin14* ^−*/*−^ mice bred on an NMRI background showed a uniform VIAAT-immunoreactivity in the LSO, whereas cochlear ablation in NMRI mice after hearing-onset did not affect the gradient. Additional analysis of C57Bl6/J, 129/SvJ and CBA/J mice revealed a strain-specific formation of the gradient.

**Conclusions:**

Our results identify an activity-regulated gradient of VIAAT in the SOC of NRMI mice. Its absence in other mouse strains adds a novel layer of strain-specific features in the auditory system, i.e. tonotopic organization of molecular gradients. This calls for caution when comparing data from different mouse strains frequently used in studies involving transgenic animals. The presence of strain-specific differences offers the possibility of genetic mapping to identify molecular factors involved in activity-dependent developmental processes in the auditory system. This would provide an important step forward concerning improved auditory rehabilitation in cases of congenital deafness.

## Background

**Fig. 1:**
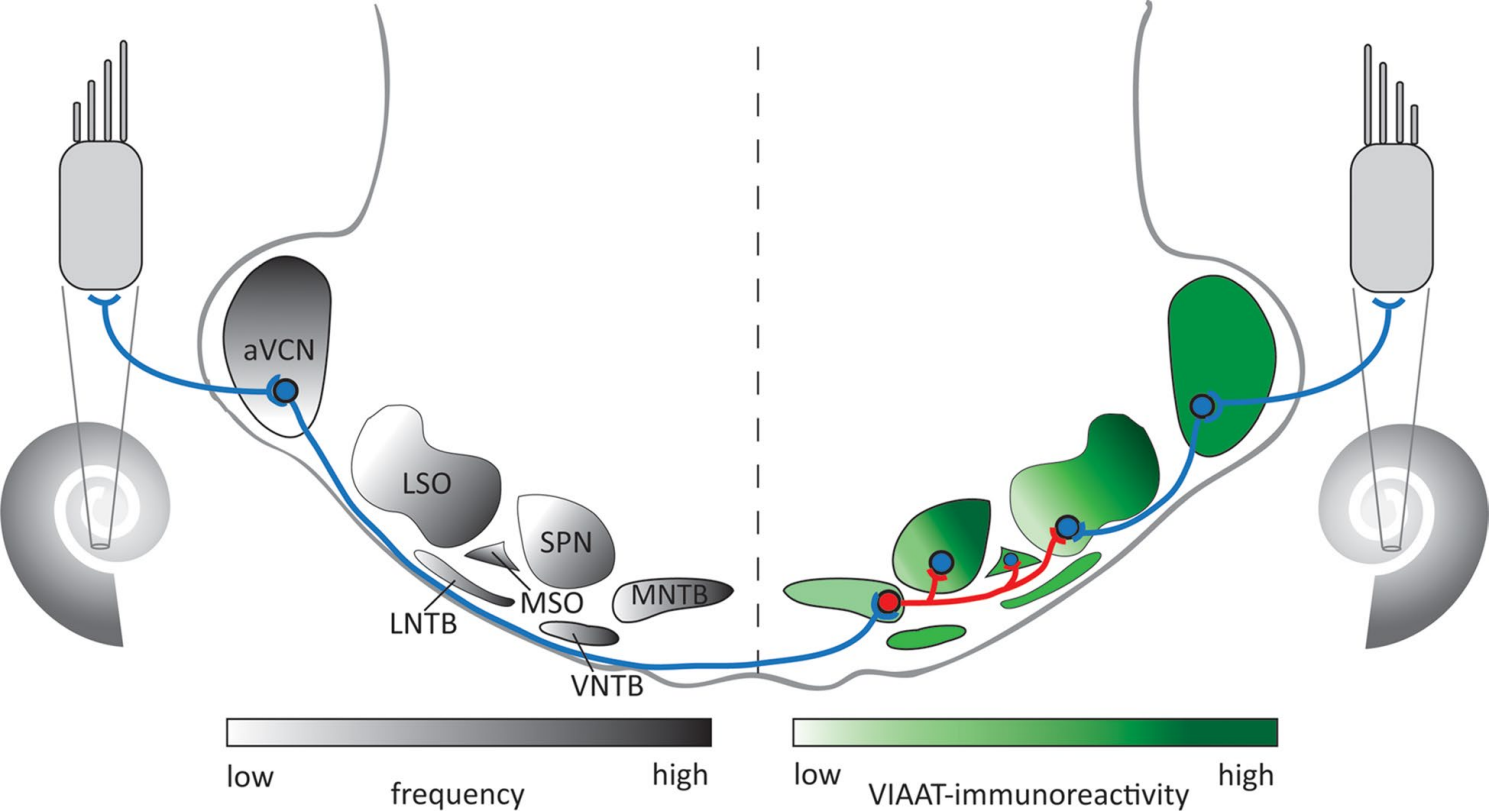
Schematic outline of auditory brainstem nuclei. Sound information is transduced in the hair cells of the cochlea and conveyed to the central auditory processing centers. The LSO is central for processing interaural level differences to detect sound sources in the horizontal plane in high frequency hearing animals. It receives excitatory input via the ipsilateral aVCN and inhibitory input via the MNTB, which in turn gets excitatory input from the contralateral aVCN. In addition, the MSO and the SPN receive inhibitory input via the MNTB. Note that not all inhibitory inputs are depicted. Gray: tonotopic gradient; green: VIAAT-immunoreactivity; blue: excitatory connection, red: inhibitory connection. aVCN: anteroventral cochlear nucleus; LNTB: lateral nucleus of the trapezoid body; LSO: lateral superior olive; MNTB: medial nucleus of the trapezoid body; MSO: medial superior olive; SPN: superior paraolivary nucleus, VNTB: ventral nucleus of the trapezoid body

The auditory brainstem of mice is composed of several nuclei organized in distinct complexes (Fig. 1). The cochlear nucleus complex (CNC) is the first station of central sound processing and consists of three nuclei: the dorsal, the posteroventral, and the anteroventral cochlear nucleus (DCN, pVCN, and aVCN, respectively). The aVCN is connected to several nuclei of the superior olivary complex (SOC; Fig. 1) [1, 2]. SOC nuclei are important for initial computation of sound source detection and sound duration [3, 4]. For both computational processes, inhibition plays an important role. In high frequency hearing animals like mice, detection of sound sources in the azimuth is mainly achieved by computing interaural level differences (ILD), arising from intensity differences at both ears [5, 6]. These differences are detected at the level of the lateral superior olive (LSO) within the SOC. To perform this task, the LSO receives excitatory input from the ipsilateral aVCN and inhibitory input from the contralateral aVCN via the ipsilateral medial nucleus of the trapezoid body (MNTB; Fig. 1). Thereby the LSO compares sound information from both ears to determine sound sources in the horizontal plane. The MNTB also provides strong inhibitory input to the medial superior olive (MSO) and the superior paraolivary nucleus (SPN, Fig. 1). In the MSO, this inhibition contributes to processing of interaural time differences [7]. In high frequency hearing animals such as the mouse, the MSO, however, plays a minor role [8]. In the SPN, inhibitory input suppresses action potential firing during a sound and triggers an offset response at sound termination [4]. This mechanism thus contributes to the encoding of duration and termination of sound, two important parameters in sensory perception.

During the analysis of various transgenic mouse models [9–12], we observed occasionally a gradient in immunoreactivity against the vesicular inhibitory amino acid transporter VIAAT (also known as VGAT) in the LSO. This protein serves as an ideal marker for inhibitory synapses, as it is the only transporter filling synaptic vesicles with the inhibitory neurotransmitters glycine and GABA [13–15]. Here, we investigated VIAAT-immunoreactivity (VIAAT-ir) in a systematic way during development in a total of six different mouse models: four different wildtype-strains, one transgenic mouse line with congenital deafness and one mouse line with cochlear ablation. This design enabled us to identify an activity-dependent and strain-specific formation of a VIAAT gradient in the SOC.

## Methods

### Animals and tissue preparation

The experiments were performed with wildtype mice of the NMRI, C57Bl6/J, 129/SvJ and CBA/J strains as well as with transgenic *Cldn14* ^−*/*−^ mice, lacking the tight junction protein claudin 14 [16]. This transgenic mouse line was bred on an NMRI background. In total 27 mice aged between postnatal days (P) 8 and 48 of both sexes were used. A detailed overview of animals is depicted in Table 1. For fixation of brain tissue, animals were anesthetized with chloral hydrate (700 mg/kg body weight i.p.) and transcardially perfused with phosphate buffered saline (PBS; 136.9 mM NaCl, 2.7 mM KCl, 10.1 mM Na_2_HPO_4_, 1.8 mM KH_2_PO_4_, pH 7.4), followed by Zamboni’s solution (2% paraformaldehyde, 15% picric acid in 0.15 M phosphate buffer). Brains were removed from the scull and postfixed for 2–4 h. in Zamboni’s solution. After incubation in 30% sucrose/PBS for cryoprotection, 30-µm-thick coronal sections were cut through the brainstem, collected in 15% sucrose/PBS, rinsed in PBS and stored at 7 °C until used for immunohistochemistry.

**Table 1:** Overview of mouse lines and number of animals used

	NMRI	C57Bl/6J	129/SvJ	CBA/J	*Cldn14* ^−*/*−^
P8	3	–	–	–	–
P16	3	–	–	–	–
P30	6	3	3	3	3
P48 (cochlea ablated)	3	–	–	–	–

### Bilateral cochlea ablation

Cochlea ablation was performed as described previously [17]. NMRI mice aged P27 were anesthetized with a mixture of Ketamine and Xylazine (90 and 7 mg/kg bodyweight i.p., respectively). The head of the animal was fixed in a stereotaxic instrument and the pinnae of both ears were tied to the front. By a deep cut behind the ear, the eardrum was uncovered and ruptured with a hollow needle to expose the auditory ossicles. After detaching the malleus, the whole organ of Corti was removed using a hollow curved needle. The wound was cleaned, filled with gel foam and surgically closed. Operated animals were administered the analgesic Novalgin (200 mg/kg bodyweight i.p.). Subsequently, animals were housed in separated cages. Weight and health conditions were checked daily. Operation was considered successful, if operated animals showed no Preyer reflex in response to sound exposure. Animals were perfused as described above 3 weeks after cochlea removal.

### Immunohistochemistry

Free floating immunohistochemistry was performed as described previously [10, 18]. Briefly, slices were incubated in blocking solution containing 3% bovine serum albumin, 10% goat serum and 0.3% Triton in PBS (pH 7.4) for 1 h at room temperature. Primary antibodies rabbit anti-VIAAT (1:500; RRID: AB_887871; Catalog No. 131002, Synaptic Systems, Göttingen, Germany), rabbit anti glycine transporter 2 (GlyT2) (1:200; RRID: AB_2619997; Catalog No. 272003, Synaptic Systems) and mouse anti-NeuN (1:1000; RRID: AB_10711040; Catalog No. ab104224, Abcam, Cambridge, UK) were diluted in carrier solution (0.3% Triton, 1% BSA, 1% goat serum in PBS, pH 7.4). Slices were incubated with primary antibody solution over night at 7 °C, rinsed in PBS and incubated in carrier solution containing an Alexa Fluor coupled secondary antibody (Alexa 488 goat anti-rabbit or Alexa 594 goat anti-mouse, 1:750, Invitrogen, Carlsbad, CA, USA) for 1.5 h at room temperature. Subsequently, slices were again rinsed in PBS, mounted on slides, air dried and cover slipped. Although, specificity of the anti-VIAAT antibody was previously shown in VIAAT knockout animals by the manufacturer (https://www.sysy.com/products/vgat/facts-131002.php), control experiments were performed by blocking the primary antibody with the corresponding control peptide (Catalog No. 131-OP, Synaptic Systems). This synthetic peptide (AEPPVEGDIHYQR) has been used for immunization. Only a faint immunosignal in the MNTB was visible after this treatment (data not shown). Specificity of NeuN and GlyT2 antibodies were tested by the manufacturer in immunohistochemistry and immunoblotting. For all primary antibodies used omission of secondary antibodies resulted in the absence of immunosignal (data not shown).

### Image acquisition

Full focus image stacks of the SOC were taken with a BZ 8100 E fluorescence microscope (Keyence, Neu-Isenburg, Germany), equipped with Nikon Plan Apo 4× and 20× objectives. A constant exposure time was set for all images. Files were obtained using the Keyence BZ observation software and processed in Adobe Photoshop CS6 (Adobe Systems) or Image J (U. S. National Institutes of Health, Bethesda, MD, USA). To be able to count VIAAT-positive synaptic boutons, detailed images of the LSO were taken with a TCS SP2 confocal microscope (Leica, Wetzlar, Germany), equipped with a Leica HCX PL APO 63× oil immersion objective. Image stacks of 1024 × 1024 pixels were taken with fixed parameters concerning laser power and pinhole settings. Images were processed using Image J.

### Gray value analysis

To analyze VIAAT-ir in the lateral and medial limb of the LSO and SPN, regions of interests (ROIs) of 70 × 70 μm or 50 × 50 µm, respectively, were analyzed for their mean gray values (3 animals per age, strain and genotype, 4–6 LSO sections per animal). Background fluorescence was determined in a third ROI outside of the SOC in a non-nucleic region. The mean value of the medial limb was set to 100%. Bars depict the relative lateral gray value compared to medial (dashed line). Using SPSS Version 23.0 (IBM Corp., Armonk, NY, USA) the data set was defined as non-Gaussian distributed. Statistical analysis was therefore performed using the Mann–Whitney *U* test. Error bars illustrate the standard deviation (SD). Analysis was carried out blind to the respective genotype.

### Bouton counts

VIAAT-positive boutons were counted in a 70 × 70 µm ROI of confocal image stacks of the lateral and medial limb of the LSO. To determine the number of VIAAT-immunoreactive boutons, images were converted into binary images and the watershed routine was applied to separate particles. Single particles were considered as boutons between a size of 0.44 and 2.20 µm as defined previously [19]. Larger particles were expected to be accumulations of single boutons. To obtain the total number of boutons, the size of accumulations was divided by 1.3 (i.e. the average bouton size). Error bars depict the SD. Student`s *t* test was used as a test for statistical significance after testing for Gaussian distribution. Analysis was carried out blind to the respective genotype.

## Results

### Developmental VIAAT-gradient in the LSO of NMRI mice

**Fig. 2:**
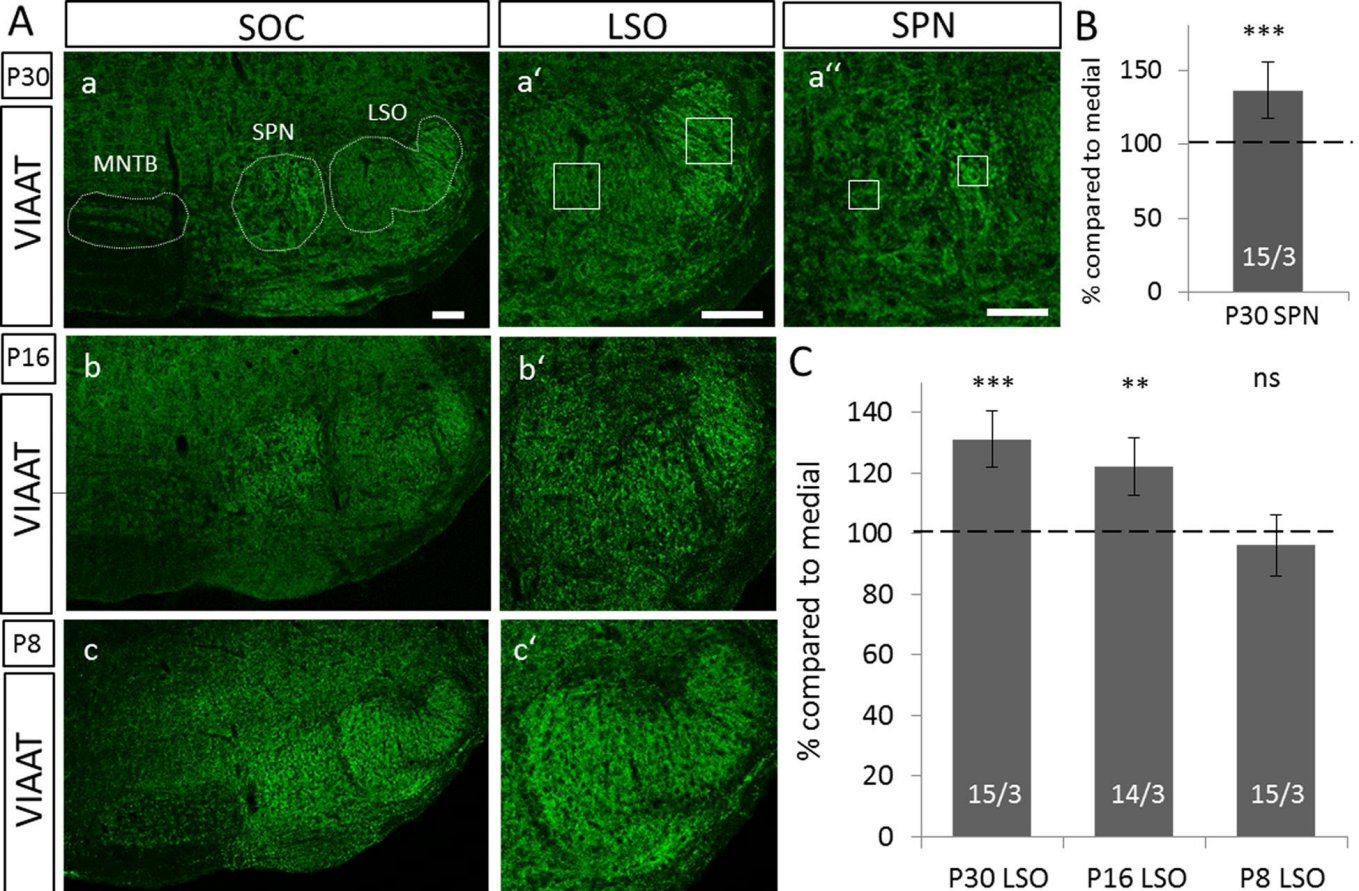
Developmental gradient of VIAAT immunoreactivity in the SOC of NMRI mice. **A**: Immunohistochemistry against VIAAT in the SOC at P30, P16 and P8 in NMRI mice. A strong gradient in immunoreactivity was found in the LSO and SPN at P30 with higher signal in lateral regions (*a*, *a*′, *a*″). This gradient was also visible at the LSO at P16, yet less pronounced (*b, b′*). No gradient was evident at P8 (*c, c′*). There was no gradient in other SOC nuclei outside the LSO and SPN (*a*, *b*). Dorsal is up, lateral to the right. Scale bars = 100 µm. **B**, **C**: Quantification of VIAAT-immunoreactivity in the SPN at P30 (**B**) and in the LSO at P30, P16 and P8 (**C**). Gray values were determined at medial and lateral regions of the respective nucleus. Bars only depict lateral values in % compared to medial. Medial values were set to 100% (dashed line). Gray values in the lateral limb of the LSO and SPN were significantly higher at P30 compared to the medial one. Significant higher immunoreactivity in the lateral LSO was already established at P16. As a test for statistical significance, Mann–Whitney *U* test was used. 14–15 ROIs were analyzed per animal and three animals per age. **p ≤ 0.05; ***p ≤ 0.001, *ns* not significant

**Fig. 3:**
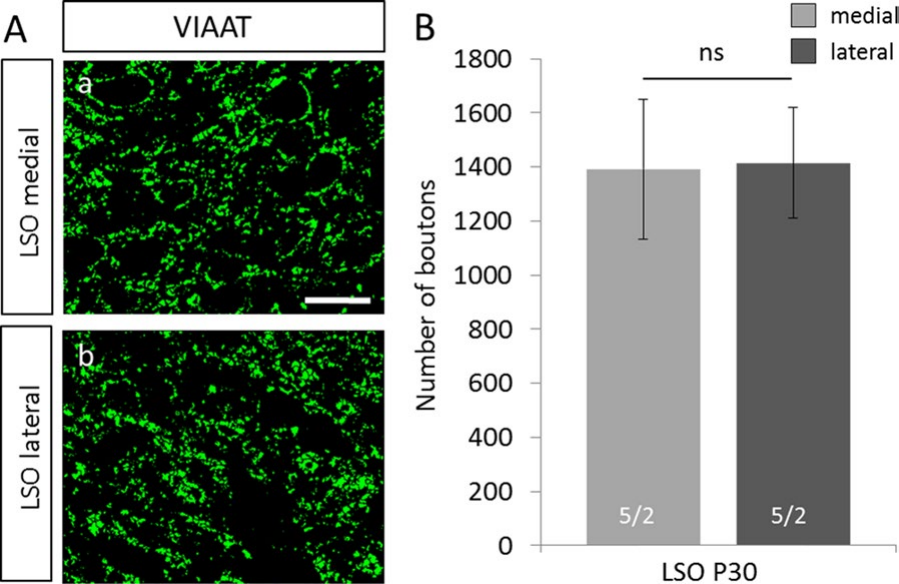
No difference in number of VIAAT-positive boutons between the lateral and medial limb of the LSO. **A**: Binary images of VIAAT immunoreactivity in the medial (*a*) and the lateral (*b*) limb of the LSO in P30 NMRI mice. Dorsal is up, lateral to the right. Scale bar = 20 µm. **B**: Quantification of VIAAT-positive boutons in the medial and lateral limb of the LSO in P30 NMRI mice. There was no significant difference in boutons number between both limbs. As a test for statistical significance, student’s *t* test was used. 5 ROIs were analyzed in each limb in 2 animals. *ns* not significant

To study inhibitory inputs in mice, we used immunoreactivity against VIAAT. This vesicular carrier serves as an important and routinely used marker for presynaptic inhibitory terminals, as it is the only transporter filling synaptic vesicles with the inhibitory neurotransmitters GABA and glycine [13–15]. In the SOC of P30 NMRI mice, VIAAT-ir showed a strong gradient across the LSO with considerably higher immunoreactivity in the lateral limb of this nucleus (Fig. 2A a, a′). To test if an increased number of boutons accounts for the strong immunosignal in the lateral limb, VIAAT-positive boutons were counted in both limbs of the LSO (Fig. 3). Quantification of VIAAT-boutons in a ROI of 70 × 70 µm revealed no difference in bouton abundancy (Fig. 3B; medial: 1392 ± 259, lateral: 1415 ± 204; *p* = 0.878), indicating a stronger VIAAT expression in synaptic boutons of the lateral limb. In addition, the SPN, which receives inhibition from the MNTB as well (Fig. 1), demonstrated a gradient in VIAAT-ir (Fig. 2A a, a″). In contrast, no gradient was obvious in other SOC nuclei. Non-auditory hindbrain regions surrounding the SOC also demonstrated a uniform VIAAT distribution (Fig. 2A a). The VIAAT-ir gradient thus appears to be specific for the LSO and SPN.

The auditory system of mice is not fully developed at birth and hearing-onset occurs around P10 [20]. We therefore investigated the time course of gradient formation. Since distinction of lateral and medial regions in the SPN is not as clear as in the LSO, most of our analysis focused on the LSO. Developmental analysis revealed that the VIAAT gradient in the LSO is mainly established between P8 and P16 (Fig. 2). While there was no visible gradient of VIAAT-ir in the LSO at P8, i.e. prior hearing-onset (Fig. 2A c, c′), it was clearly visible at P16, which is about 4 days after hearing-onset (Fig. 2A b, b′). The gradient became even more pronounced at P30 (Fig. 2A a, a′). Quantification of VIAAT-immunosignal confirmed this observation (Fig. 2C). Gray values of the lateral limb were 22% higher in P16 animals (medial: 1.453 ± 0.158, lateral: 1.770 ± 0.198; *p* = 0.001) and 31% at P30 (medial: 1.298 ± 0.123, lateral: 1.702 ± 0.204, *p* = 0.000012) compared to the medial limb. Thus, the VIAAT-ir gradient forms during postnatal development around hearing-onset. Additional quantification of the VIAAT-ir in lateral and medial regions of the SPN at P30 revealed a 36% higher VIAAT-ir in lateral regions of this nucleus (Fig. 2B, medial: 1.925 ± 0.379, lateral: 2.579 ± 0.346, *p* = 0.000071).

**Fig. 4:**
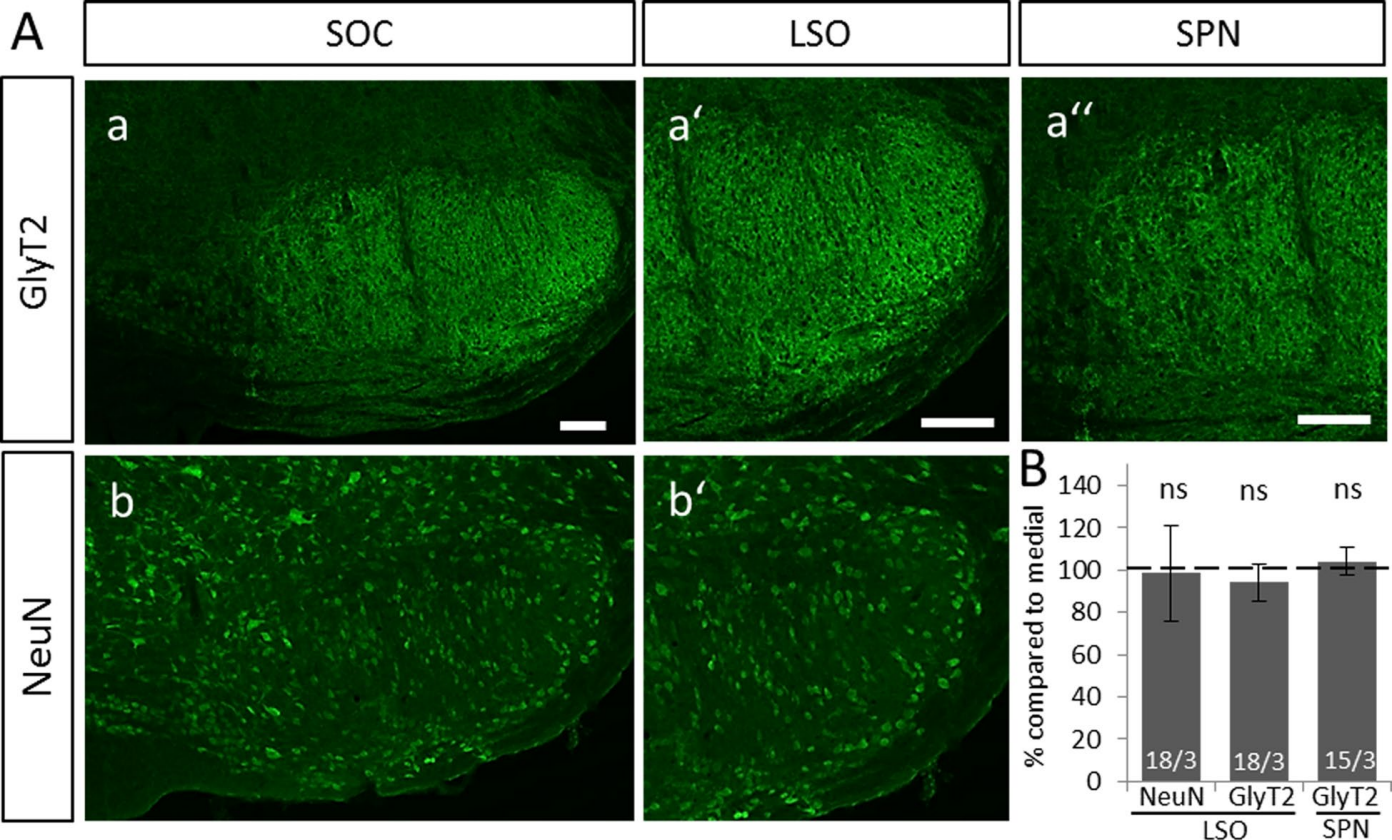
No gradient in immunoreactivity of other marker proteins in the SOC of NMRI mice. **A**: Immunohistochemistry against the inhibitory marker GlyT2 (*a*) and the neuronal marker NeuN (*b*) showed no obvious gradient in the LSO (*a′, b′*) or SPN (*a″*) of NMRI mice at P30. **B**: Quantification of gray values in the LSO and SPN of NMRI mice at P30 based on GlyT2 and NeuN immunoreactivity revealed no difference between lateral and medial regions in either nucleus. Bars only depict lateral values in % compared to medial. Medial values were set to 100% (dashed line). Statistical significance was tested using the Mann–Whitney *U* test. 15–18 ROIs were analyzed per animal and three animals per age. *ns* not significant

To control for the possibility of a general gradient of neuronal or inhibitory proteins in the LSO and SPN of NMRI mice, we counterstained these nuclei with the neuronal marker NeuN and GlyT2, a marker for glycinergic synapses (Fig. 4A). Quantification of gray values in medial and lateral regions revealed no gradient in either nucleus (Fig. 4B, LSO NeuN medial: 1.482 ± 0.224, LSO NeuN lateral: 1.504 ± 0.604, *p* = 0.628; LSO GlyT2 medial: 2.839 ± 0.549, LSO GlyT2 lateral: 2.678 ± 0.604, *p* = 0.239; SPN GlyT2 medial: 2.654 ± 0.524, SPN GlyT2 lateral: 2.742 ± 0.425, *p* = 0.367). Since it was not possible to clearly delineate the SPN in NeuN immunoreactive slices, only values for GlyT2 were calculated in this nucleus.

### Activity-dependent formation of the VIAAT-gradient in the NMRI strain

**Fig. 5:**
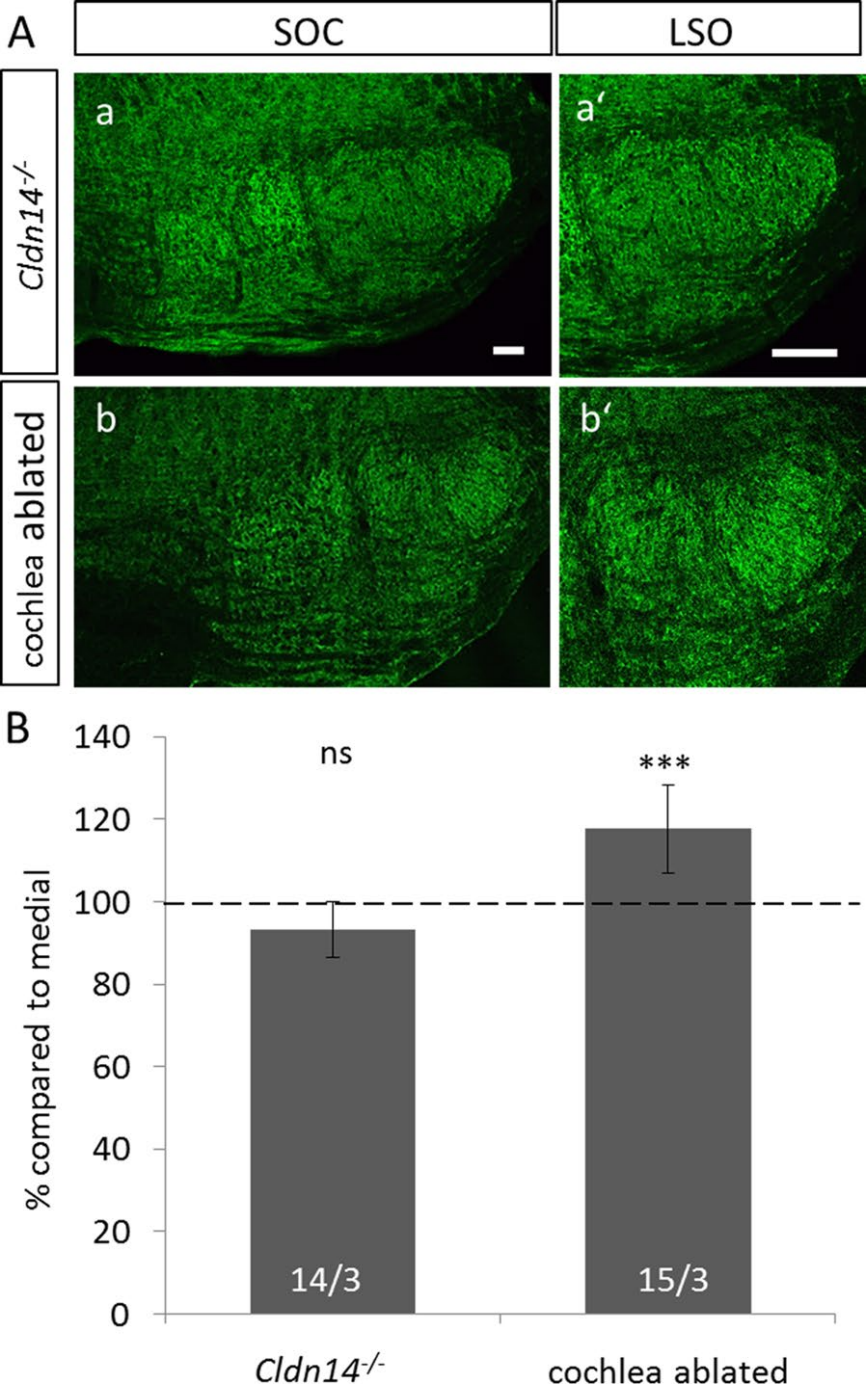
Loss of auditory sensory activity before but not after onset of hearing influences the formation of the VIAAT-gradient in NMRI mice. **A**: No gradient in VIAAT-immunoreactivity was evident in SOC nuclei of deaf *Cldn14* ^−*/*−^ mice at P30 (*a, a′*). However, in animals subjected to bilateral cochlea ablation 2 weeks after hearing-onset, a clear gradient in the LSO was visible (*b′*). **B**: Quantification of VIAAT immunoreactivity in deaf and cochlea ablated animals. There was no difference between the medial and the lateral limb of the LSO in the congenital deaf mouse line but a significant higher immunoreactivity in the lateral limb of cochlea ablated animals. Bars only depict lateral values in % compared to medial. Medial values were set to 100% (dashed line). Statistical significance was tested using the Mann–Whitney *U* test. 14–15 ROIs were analyzed per animal and three animals per age. ***p ≤ 0.001, *ns* not significant

The gradient in VIAAT-ir in NMRI mice is established during postnatal development of the auditory brainstem between P8 and P16. During this time period, neural activity along the auditory pathway can influence developmental processes [19, 21–24]. To investigate the role of neuronal activity in gradient formation and maintenance, we first investigated *Cldn14* ^−*/*−^ mice. These mice lack the tight junction protein claudin 14 and display a normal development of the auditory system until their hair cells degenerate before hearing-onset [16]. To test whether this affects formation of the VIAAT-ir gradient, *Cldn14* ^−*/*−^ mice bred on an NMRI background were subjected to quantitative VIAAT-ir (Fig. 5A a, a′). We found no significant differences in intensity between the lateral and the medial limb of the LSO in these mice (Fig. 5B; medial: 1.449 ± 0.125, lateral: 1.351 ± 0.217, *p* = 0.108). This result reveals that formation of the gradient requires neuronal activity.

To investigate whether also maintenance of the gradient is activity-dependent, we analyzed mice with cochlear ablation after hearing-onset. To this end, NMRI mice were subjected to bilateral cochlea removal at P27, i.e. roughly 2 weeks after hearing-onset. Successfully operated animals were killed 3 weeks after deafening at P48. Subsequent anti-VIAAT immunohistochemistry revealed the persistence of the gradient in these mice (Fig. 5A b, b′). There was a significant 18% upregulation of the immunosignal in the lateral limb compared to the medial one (Fig. 5B; medial: 1.176 ± 0.115, lateral: 1.379 ± 0.122. *p* = 0.000242). This result indicates that activity deprivation after hearing-onset does not influence the VIAAT gradient, at least during a 3-week period. Taken together, these results reveal that neuronal activity is required for the formation of the VIAAT-ir gradient in the LSO, but not for its maintenance.

### No VIAAT-gradient in other wildtype mouse strains

**Fig. 6:**
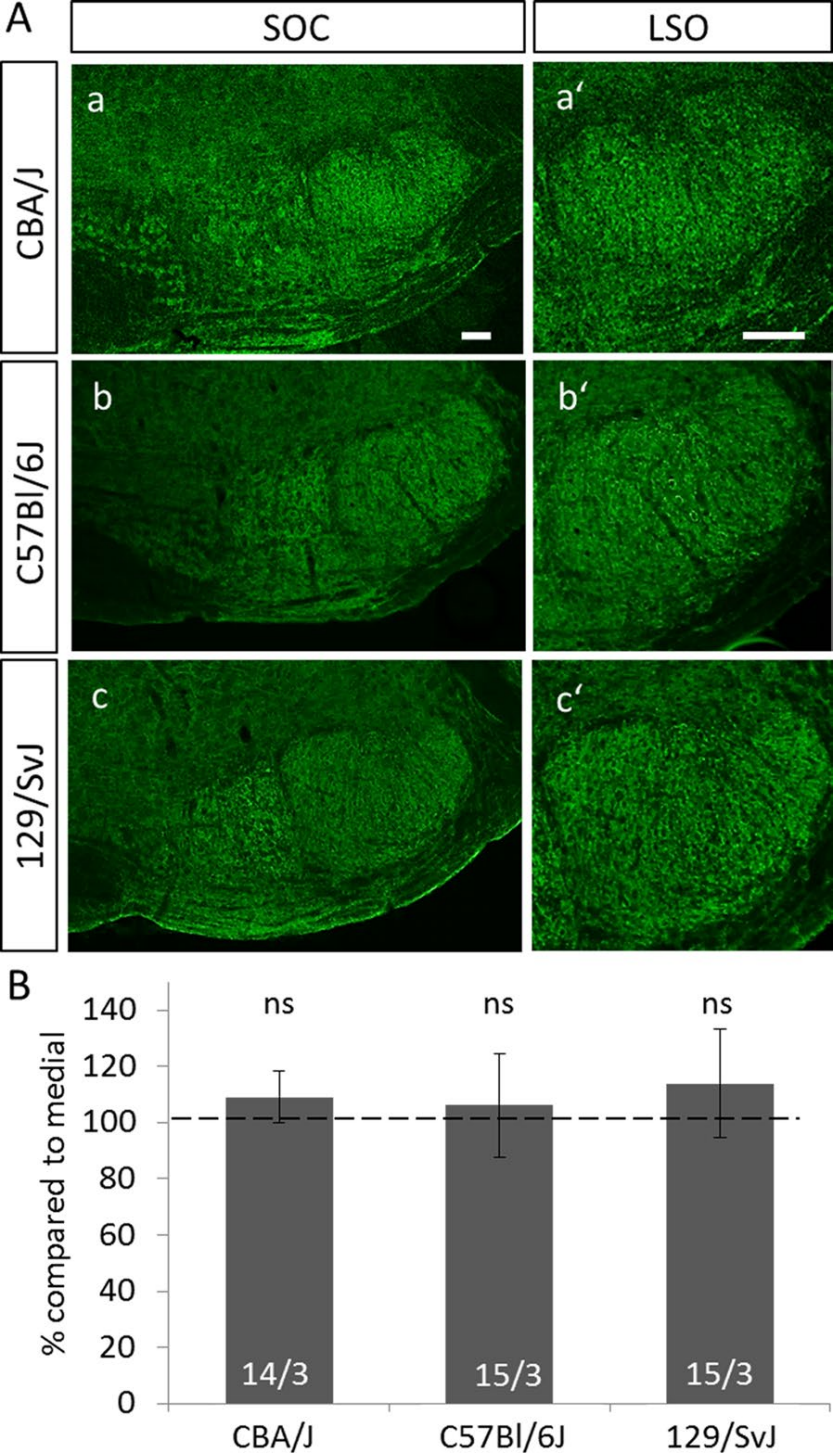
No VIAAT-gradient in different inbred mouse strains. **A**: No gradient in VIAAT-immunoreactivity was evident in the SOC of P30 mice of the CBA/J (*a, a′*), C57Bl/6J (*b, b′*) and the 129/SvJ (*c, c′*) strain. **B**: Quantification of gray values of the VIAAT-immunoreactivity in the medial and lateral limb of the LSO of CBA/J, C57Bl/6J and 129/SvJ mice revealed no differences in immunosignal between the two limbs. Bars only depict lateral values in % compared to medial. Medial values were set to 100% (dashed line). Statistical significance was tested using the Mann–Whitney *U* test. 14–15 ROIs were analyzed per animal and three animals per age. *ns* not significant

Previous studies in our group [10–12, 19] led to the impression that the presence of the gradient was variable in control animals. We therefore hypothesized that it might be a function of the mouse strain in use. To test this conjecture, we also quantified VIAAT-ir in the LSO of three additional wildtype strains, namely CBA/J, C57Bl/6J and 129/SvJ (Fig. 6A). Again, the quantitative analysis focused only on the LSO. Comparing the gray values of VIAAT-labeling in the lateral and the medial limb of the LSO at P30 in these strains revealed no significant differences (Fig. 6B; CBA/J medial: 1.752 ± 0.199, CBA/J lateral: 1.904 ± 0.234 *p* = 0.108; C57Bl/6J medial: 1.921 ± 0.347, C57Bl/6J lateral: 2.023 ± 0.434, *p* = 0.73; 129/SvJ medial: 1.827 ± 0.471, 129/SvJ lateral: 2.056 ± 0.568, *p* = 0.12). This finding suggests that the gradient of VIAAT in the LSO of NMRI mice is strain-specific. Furthermore, no gradient was obvious in the SPN.

## Discussion

Here, we report a gradient of VIAAT-ir in the LSO and SPN of NMRI mice with high expression in the lateral limb and lower expression in the medial limb. This VIAAT-gradient does not result from an increased bouton number in the lateral limb compared to the medial one as shown by quantification of boutons in the LSO (Fig. 3). The constant number of VIAAT-positive boutons in both limbs of the LSO in NMRI mice suggests a higher expression of VIAAT in single boutons of the medial limb. This can be explained by either an increased number of transporters per vesicle or by a larger vesicle pool in lateral boutons.

### Tonotopic organization of the auditory system

Overall, the gradient followed the medio-lateral axis of the LSO and SPN and thus their tonotopic organization as low frequencies are processed in the lateral and high frequencies in the medial part of these nuclei. Tonotopic organization, i.e. the tuning of topologically neighboring neurons to neighboring frequencies, is a characteristic property of the auditory system (Fig. 1). Matching this tonotopic organization, several molecular gradients in expression patterns have been observed in the auditory system. In the cochlea, there is an opposing gradient of *Kcnq4*-expression, coding for the voltage gated potassium channel K_v_7.4, in inner and outer hair cells [25, 26]. Additionally, the calcium binding proteins calbindin and β-parvalbumin display opposing gradients along the cochlea [27, 28]. On the level of the auditory brainstem, gradients of the potassium channels K_v_1.1 and K_v_1.3 are present in MNTB and LSO [29–31]. In the LSO of rats and gerbils there is a medio-lateral gradient of the glycine receptor with a higher abundancy in the medial limb [32–34]. Additionally, stronger immunoreactivity against the inhibitory neurotransmitters glycine and GABA was found in the medial limb of the LSO in gerbils, although this is most likely attributed to a higher neuron number in this limb [35]. So far, no gradient in the SPN was reported.

Presumably, these gradients provide the molecular basis for an efficient transduction of high frequencies in the medial limb of the auditory nuclei as fast repolarization and a reliable inhibition is crucial for the computation of high frequency sounds [36, 37]. Of note, the here reported VIAAT gradient shows strongest labeling in the lateral limb, which contrasts the previously described gradients of proteins associated with inhibitory transmission in gerbils and rats, which show high labeling of glycine receptors in the medial limb [32–34]. The physiological relevance of these inverse oriented gradients has still to be elucidated.

### Activity-dependent formation of tonotopic gradients

The formation of tonotopic gradients often depend on the interplay between molecular factors and neuronal activity [38, 39]. Our analysis revealed that neuronal activity is required for the formation of this gradient, as the gradient is disrupted in deaf *Cldn14* ^−*/*−^ mice. The appearance of the gradient between P8 and P16 indicates that its formation requires sound-driven activity. Furthermore, spontaneous activity is likely present in the *Cldn14* ^−*/*−^ mouse model, as hair cells appear normal up to P7, and rapid loss of outer hair cells begins only thereafter [16]. However, a recent analysis of mice lacking the α9 subunit of nicotinic acetylcholine receptors demonstrated that already subtle alterations in the temporal fine structure of spontaneous activity disrupt proper development of the auditory brainstem such as correct axonal pruning [40]. To distinguish between the role of spontaneous versus sensory evoked activity in formation of the gradient, we attempted cochlear ablations around hearing-onset. However, initial experiments yielded poor survival rates. Thus, further studies are required to firmly conclude that the gradient depends solely on sensory-evoked activity.

Activity-dependent formation of tonotopic features agrees well with a previous analysis of tonotopic features in MNTB neurons of wildtype or congenital deaf *dn/dn* mice. Electrophysiological and molecular investigations in wildtype mice revealed gradients in high-and low threshold potassium conductances as well as in immunoreactivity for K_v_3.1 and K_v_1.1, contributing to these conductances. These tonotopic gradients were abrogated in congenital deaf *dn/dn* mice [31]. A similar observation was made for HNC4 channels and the associated hyperpolarization-activated currents [31]. Furthermore, the non-uniform distribution of glycine receptors in the LSO of gerbils is not established in cochlea ablated animals [41]. Finally, a recent analysis revealed an activity-dependent formation of a neuronal size gradient of MNTB neurons [42].

Sensory activity is not only required for proper formation but also for maintenance of molecular patterns. A genome wide microarray analysis performed 3 or 21 days after cochlear ablation in adult rats detected 449 or 1.031 genes, respectively, with altered expression pattern. Among them were many genes encoding proteins for neurotransmission such as SNAP25 or GABA receptors [43]. As this study indicated stronger impact 3 weeks after sensory deprivation, we probed the role of activity for maintenance 21 days after cochlear ablation. This experimental design, however, did not interfere with the presence of the gradient. Therefore, sensory activity is apparently not required for the maintenance of the VIAAT-gradient once it has been established.

### Strain-specific formation of the VIAAT-ir gradient

Intriguingly, the gradient was only present in NMRI mice among four different mouse strains tested (Fig. 2, 6). Noteworthy, this is to our knowledge the first time of a mouse strain-specific feature in tonotopicity. Well-established strain-specific auditory features refer to age-related hearing loss or differences in auditory brainstem responses [44–46]. A splice variant of *cadherin* 23 which causes early onset of hearing loss after ~ 13 weeks of age is only present in a subset of mouse strains [47, 48]. Cadherin 23 was shown to be involved in tip-link formation between stereocilia of cochlear hair cells [49]. With respect to the central auditory pathway, detailed analysis of auditory brainstem responses revealed variation between 10 tested mouse strains. This concerned the amplitude increase with intensity, which reached a maximum and then leveled off or decreased with higher intensity, inter-peak I–IV latencies or the slope of latency–intensity function [46]. Unfortunately, NMRI mice had not been included in this study. A previous comparison of hearing thresholds in C57Bl/6J and NMRI mice at 7 month of age showed good hearing ability in the NMRI strain [50]. The good hearing of NMRI mice is also reflected in the frequent use of this strain in behavioral auditory studies [51–55]. However, to our knowledge there is no known characteristic in the hearing ability of NMRI mice that could account for the observed VIAAT-gradient in the LSO and SPN of these animals.

Finally, strain-specific formation of the VIAAT-gradient will provide a powerful tool for the identification of genetic factors involved in activity-dependent processes in the auditory system. This is of great importance in the auditory field, as deafness is among the most common sensory disorders [56, 57]. Cochlear implants are available to restore peripheral dysfunction, but do not bypass central auditory defects, which frequently occur [58].

One possibility is quantitative trait locus mapping [59]. This technique was already successfully used to identify a mutation in *cadherin 23* associated with age-related hearing loss [60]. Recent advances such as the availability of chromosome substitution strains further alleviate this approach [61]. Alternatively, comparative transcriptome analysis can be performed between the SOC of NMRI mice and C57Bl6/J to identify genetic factors. A similar approach had recently identified crystallin N as an important protein for proper auditory brainstem development [12, 62]. A strain-based approach will be superior to the comparison of normal hearing and deaf mice, as the differences in gene expression are likely subtler between strains, facilitating the identification of candidate genes.

## Conclusion

In this study, we reported a higher VIAAT-ir in lateral regions of the LSO and SPN compared to medial regions. The establishment of this gradient is developmentally regulated and forms between P8 and P16 in NMRI mice. Analysis of other commonly used wildtype strains revealed no such gradient, suggesting a strain-specific characteristic. This specificity paves the way to identify genetic factors involved in activity-dependent processes in the auditory hindbrain. This will be important with respect to auditory rehabilitation. Congenital deafness will interrupt those processes, therefore impairing the benefit from cochlear implants. Currently, the molecular mechanisms governing activity-dependent processes in the auditory system are unknown, thereby preventing new therapeutic approaches for restoring central auditory defects. Furthermore, our findings extend the auditory repertoire of strain-specific features, thus sending a note of caution when comparing data from different mouse strains.

## Abbreviations

aVCN: Anteroventral cochlear nucleus complex
CNC: Cochlear nucleus complex
DCN: Dorsal cochlear nucleus complex
GlyT2: Glycine transporter 2
ILD: Interaural level differentiation
Ir: Immunoreactivity
LSO: Lateral superior olive
MNTB: Medial nucleus of the trapezoid body
MSO: Medial superior olive
P: Postnatal day
pVCN: Posteroventral cochlear nucleus complex
ROI: Region of interest
SD: Standard deviation
SOC: Superior olivary complex
SPN: Superior paraolivary nucleus
VIAAT: Vesicular inhibitory amino acid transporter

## References

[1] Moore DR (1991). Anatomy and physiology of binaural hearing. Audiology.

[2] Thompson AM, Schofield BR (2000). Afferent projections of the superior olivary complex. Microsc Res Tech.

[3] Kadner A, Kulesza R, Berrebi A (2006). Neurons in the medial nucleus of the trapezoid body and superior paraolivary nucleus of the rat may play a role in sound duration coding. J Neurophysiol.

[4] Kopp-Scheinpflug C, Tozer AJ, Robinson SW, Tempel BL, Hennig MH, Forsythe ID (2011). The sound of silence: ionic mechanisms encoding sound termination. Neuron.

[5] Grothe B (2003). New roles for synaptic inhibition in sound localization. Nat Rev Neurosci.

[6] Grothe B, Pecka M (2014). The natural history of sound localization in mammals—a story of neuronal inhibition. Front Neural Circuits.

[7] Brand A, Behrend O, Marquardt T, McAlpine D, Grothe B (2002). Precise inhibition is essential for microsecond interaural time difference coding. Nature.

[8] Grothe B, Pecka M, McAlpine D (2010). Mechanisms of sound localization in mammals. Physiol Rev.

[9] Hirtz JJ, Boesen M, Braun N, Deitmer JW, Kramer F, Lohr C (2011). Cav1.3 calcium channels are required for normal development of the auditory brainstem. J Neurosci.

[10] Ebbers L, Satheesh SV, Janz K, Rüttiger L, Blosa M, Hofmann F (2015). L-type calcium channel Cav1.2 is required for maintenance of auditory brainstem nuclei. J Biol Chem.

[11] Satheesh S, Kunert K, Rüttiger L, Zuccotti A, Schönig K, Friauf E (2012). Retrocochlear function of the peripheral deafness gene Cacna1d. Hum Mol Genet.

[12] Hartwich H, Rosengauer E, Rüttiger L, Wilms V, Waterholter S-KK, Nothwang HG (2016). Functional role of γ-crystallin N in the auditory hindbrain. PLoS ONE.

[13] McIntire SL, Reimer RJ, Schuske K, Edwards RH, Jorgensen EM (1997). Identification and characterization of the vesicular GABA transporter. Nature.

[14] Sagné C, El Mestikawy S, Isambert MF, Hamon M, Henry JP, Giros B (1997). Cloning of a functional vesicular GABA and glycine transporter by screening of genome databases. FEBS Lett.

[15] Wojcik SM, Katsurabayashi S, Guillemin I, Friauf E, Rosenmund C, Brose N (2006). A shared vesicular carrier allows synaptic corelease of GABA and glycine. Neuron.

[16] Ben-Yosef T, Belyantseva I, Saunders T, Hughes E, Kawamoto K, Itallie C (2003). Claudin 14 knockout mice, a model for autosomal recessive deafness DFNB29, are deaf due to cochlear hair cell degeneration. Hum Mol Genet.

[17] Illing RB, Horváth M, Laszig R (1997). Plasticity of the auditory brainstem: effects of cochlear ablation on GAP-43 immunoreactivity in the rat. J Comp Neurol.

[18] Ebbers L, Runge K, Nothwang HG (2016). Differential patterns of histone methylase EHMT2 and its catalyzed histone modifications H3K9me1 and H3K9me2 during maturation of central auditory system. Cell Tissue Res.

[19] Hirtz JJ, Braun N, Griesemer D, Hannes C, Janz K, Löhrke S (2012). Synaptic refinement of an inhibitory topographic map in the auditory brainstem requires functional Cav1.3 calcium channels. J Neurosci.

[20] Mikaelian D, Ruben R (1965). Development of hearing in the normal Cba-J mouse: correlation of physiological observations with behavioral responses and with cochlear anatomy. Acta Oto-laryngol.

[21] Sanes D, Takács C (1993). Activity-dependent refinement of inhibitory connections. Eur J Neurosci.

[22] Kapfer C, Seidl A, Schweizer H, Grothe B (2002). Experience-dependent refinement of inhibitory inputs to auditory coincidence-detector neurons. Nat Neurosci.

[23] Noh J, Seal RP, Garver JA, Edwards RH, Kandler K (2010). Glutamate co-release at GABA/glycinergic synapses is crucial for the refinement of an inhibitory map. Nat Neurosci.

[24] Kandler K, Gillespie D (2005). Developmental refinement of inhibitory sound-localization circuits. Trends Neurosci.

[25] Beisel K, Nelson N, Delimont D, Fritzsch B (2000). Longitudinal gradients of KCNQ4 expression in spiral ganglion and cochlear hair cells correlate with progressive hearing loss in DFNA211Published on the World Wide Web on 13 September 2000. Mol Brain Res.

[26] Beisel K, Rocha-Sanchez S, Morris K, Nie L, Feng F, Kachar B (2005). Differential expression of KCNQ4 in inner hair cells and sensory neurons is the basis of progressive high-frequency hearing loss. J Neurosci.

[27] Pack AK, Slepecky NB (1995). Cytoskeletal and calcium-binding proteins in the mammalian organ of Corti: cell type-specific proteins displaying longitudinal and radial gradients. Hear Res.

[28] Hackney C, Mahendrasingam S, Penn A, Fettiplace R (2005). The concentrations of calcium buffering proteins in mammalian cochlear hair cells. J Neurosci.

[29] Barnes-Davies M, Barker M, Osmani F, Forsythe I (2004). Kv1 currents mediate a gradient of principal neuron excitability across the tonotopic axis in the rat lateral superior olive. Eur J Neurosci.

[30] Li W, Kaczmarek L, Perney T (2001). Localization of two high threshold potassium channel subunits in the rat central auditory system. J Comp Neurol.

[31] Leao R, Sun H, Svahn K, Berntson A, Youssoufian M, Paolini A (2006). Topographic organization in the auditory brainstem of juvenile mice is disrupted in congenital deafness. J Physiol.

[32] Friauf E, Hammerschmidt B, Kirsch J (1997). Development of adult-type inhibitory glycine receptors in the central auditory system of rats. J Comp Neurology.

[33] Sanes DH, Wooten GF (1987). Development of glycine receptor distribution in the lateral superior olive of the gerbil. J Neurosci.

[34] Sanes DH, Geary WA, Wooten GF, Rubel EW (1987). Quantitative distribution of the glycine receptor in the auditory brain stem of the gerbil. J Neurosci.

[35] Gleich O, Weiss M, Strutz J (2004). Age-dependent changes in the lateral superior olive of the gerbil (*Meriones unguiculatus*). Hear Res.

[36] Caspary DM (2001). Focus: GABA and glycine neurotransmission in mouse auditory brainstem structures.

[37] Brew HM, Forsythe ID (2005). Systematic variation of potassium current amplitudes across the tonotopic axis of the rat medial nucleus of the trapezoid body. Hear Res.

[38] Friauf Lohmann C (1999). Development of auditory brainstem circuitry. Cell Tissue Res.

[39] Nothwang HG, Ebbers L, Schlüter T, Willaredt MA (2015). The emerging framework of mammalian auditory hindbrain development. Cell Tissue Res.

[40] Clause A, Kim G, Sonntag M, Weisz CJ, Vetter DE, Rűbsamen R (2014). The precise temporal pattern of prehearing spontaneous activity is necessary for tonotopic map refinement. Neuron.

[41] Koch U, Sanes DH (1998). Afferent regulation of glycine receptor distribution in the gerbil LSO. Microsc Res Tech.

[42] Weatherstone JH, Kopp-Scheinpflug C, Pilati N, Wang Y, Forsythe ID, Rubel EW, et al. Maintenance of neuronal size gradient in MNTB requires sound-evoked activit. J. Neurophysiol. 2016;jn.00528.2016. 10.1152/jn.00528.2016 PMC5304411 27881722

[43] Holt AG, Asako M, Lomax CA, MacDonald JW, Tong L, Lomax MI (2005). Deafness-related plasticity in the inferior colliculus: gene expression profiling following removal of peripheral activity. J Neurochem.

[44] Kane KL, Longo-Guess CM, Gagnon LH, Ding D, Salvi RJ, Johnson KR (2012). Genetic background effects on age-related hearing loss associated with Cdh23 variants in mice. Hear Res.

[45] Johnson K, Zheng Q, Noben-Trauth K (2006). Strain background effects and genetic modifiers of hearing in mice. Brain Res.

[46] Zhou X, Jen PH, Seburn KL, Frankel WN, Zheng QY (2006). Auditory brainstem responses in 10 inbred strains of mice. Brain Res.

[47] Noben-Trauth K, Zheng QY, Johnson KR (2003). Association of cadherin 23 with polygenic inheritance and genetic modification of sensorineural hearing loss. Nat Genet.

[48] Zheng Q, Johnson K, Erway L (1999). Assessment of hearing in 80 inbred strains of mice by ABR threshold analyses. Hear Res.

[49] Kazmierczak P, Sakaguchi H, Tokita J, Wilson-Kubalek E, Milligan R, Müller U (2007). Cadherin 23 and protocadherin 15 interact to form tip-link filaments in sensory hair cells. Nature.

[50] Heffner RS, Koay G, Heffner HE. Focus: Sound localization acuity changes with age in C57Bl/6J mice. 2001. p. 31–35.

[51] Kurt S, Ehret G (2010). Auditory discrimination learning and knowledge transfer in mice depends on task difficulty. P Natl Acad Sci USA.

[52] Ehret G (1975). Frequency and intensity difference limens and nonlinearities in the ear of the housemouse (*Mus musculus*). J Comp Physiol.

[53] Ehret G (1975). Masked auditory thresholds, critical ratios, and scales of the basilar membrane of the housemouse (*Mus musculus*). J Comp Physiol.

[54] Ehret G (1976). Temporal auditory summation for pure tones and white noise in the house mouse (*Mus musculus*). J Acoust Soc Am.

[55] Klink KB, Klump GM (2004). Duration discrimination in the mouse (*Mus musculus*). J Comp Physiol A Neuroethol Sens Neural Behav Physiol..

[56] Raviv D, Dror AA, Avraham KB (2010). Hearing loss: a common disorder caused by many rare alleles. Ann N Y Acad Sci.

[57] Angeli S, Lin X, Liu XZ (2012). Genetics of hearing and deafness. Anat Rec (Hoboken).

[58] Willaredt MA, Ebbers L, Nothwang HG (2014). Central auditory function of deafness genes. Hear Res.

[59] Armstrong NJ, Brodnicki TC, Speed TP (2006). Mind the gap: analysis of marker-assisted breeding strategies for inbred mouse strains. Mamm Genome.

[60] Johnson KR, Zheng QY, Erway LC (2000). A major gene affecting age-related hearing loss is common to at least ten inbred strains of mice. Genomics.

[61] Nadeau JH, Forejt J, Takada T, Shiroishi T (2012). Chromosome substitution strains: gene discovery, functional analysis, and systems studies. Mamm Genome.

[62] Ehmann H, Hartwich H, Salzig C, Hartmann N, Clément-Ziza M, Ushakov K (2013). Time-dependent gene expression analysis of the developing superior olivary complex. J Biol Chem.

